# Estimating heterogeneity of physical function treatment response to caloric restriction among older adults with obesity

**DOI:** 10.1371/journal.pone.0267779

**Published:** 2022-05-05

**Authors:** Daniel P. Beavers, Katherine L. Hsieh, Dalane W. Kitzman, Stephen B. Kritchevsky, Stephen P. Messier, Rebecca H. Neiberg, Barbara J. Nicklas, W. Jack Rejeski, Kristen M. Beavers

**Affiliations:** 1 Department of Biostatistics and Data Science, Wake Forest School of Medicine, Winston-Salem, NC, United States of America; 2 Section on Gerontology and Geriatric Medicine, Wake Forest School of Medicine, Winston-Salem, NC, United States of America; 3 Section on Cardiovascular Medicine, Wake Forest School of Medicine, Winston-Salem, NC, United States of America; 4 Department of Health and Exercise Science, Wake Forest University, Winston-Salem, NC, United States of America; Instituto Nacional de Geriatria, MEXICO

## Abstract

Clinical trials conventionally test aggregate mean differences and assume homogeneous variances across treatment groups. However, significant response heterogeneity may exist. The purpose of this study was to model treatment response variability using gait speed change among older adults participating in caloric restriction (CR) trials. Eight randomized controlled trials (RCTs) with five- or six-month assessments were pooled, including 749 participants randomized to CR and 594 participants randomized to non-CR (NoCR). Statistical models compared means and variances by CR assignment and exercise assignment or select subgroups, testing for treatment differences and interactions for mean changes and standard deviations. Continuous equivalents of dichotomized variables were also fit. Models used a Bayesian framework, and posterior estimates were presented as means and 95% Bayesian credible intervals (BCI). At baseline, participants were 67.7 (SD = 5.4) years, 69.8% female, and 79.2% white, with a BMI of 33.9 (4.4) kg/m^2^. CR participants reduced body mass [CR: -7.7 (5.8) kg vs. NoCR: -0.9 (3.5) kg] and increased gait speed [CR: +0.10 (0.16) m/s vs. NoCR: +0.07 (0.15) m/s] more than NoCR participants. There were no treatment differences in gait speed change standard deviations [CR–NoCR: -0.002 m/s (95% BCI: -0.013, 0.009)]. Significant mean interactions between CR and exercise assignment [0.037 m/s (95% BCI: 0.004, 0.070)], BMI [0.034 m/s (95% BCI: 0.003, 0.066)], and IL-6 [0.041 m/s (95% BCI: 0.009, 0.073)] were observed, while variance interactions were observed between CR and exercise assignment [-0.458 m/s (95% BCI: -0.783, -0.138)], age [-0.557 m/s (95% BCI: -0.900, -0.221)], and gait speed [-0.530 m/s (95% BCI: -1.018, -0.062)] subgroups. Caloric restriction plus exercise yielded the greatest gait speed benefit among older adults with obesity. High BMI and IL-6 subgroups also improved gait speed in response to CR. Results provide a novel statistical framework for identifying treatment heterogeneity in RCTs.

## Introduction

Clinical trials with continuous outcomes are conventionally designed to test aggregate mean differences, typically assuming homogeneous variances across treatment groups. While useful in guiding overall recommendations, this approach often fails to uncover prognostic indicators or underlying mechanisms of treatment effects. Indeed, experiential wisdom imparts that intervention efficacy can vary significantly by subgroup [1, 2], with individualized clinical decision making a fundamental tenet of precision medicine [3]. In pharmaceutical trials, for example, identification of “high responders” has led to prescribing practices aimed at maximizing treatment benefit while minimizing side effects [4, 5]. Consideration of individualized response in lifestyle-based trials has also helped to identify subgroups that are more likely to respond to exercise [6, 7] and nutrition [8, 9] prescriptions designed to mitigate chronic disease risk.

While quantifying response variability is generally recognized as important [10], it can be technically challenging. For instance, variability often stems from within-subject characteristics [11, 12] which can be difficult to separate from random error [13]. Additionally, many trials are not designed with sufficient power to adequately examine mean subgroup differences; and attempts to do so rarely employ appropriate methodology to test for heterogeneity of variances. Assumed homogeneous variances can be problematic if they lead to biased variance estimates—negatively impacting analytic power, probabilities associated with individual response, and understanding of within-subgroup treatment effects. Furthermore, existing statistical methods are most appropriate for parallel group studies [14]; thus, the ability to test for treatment heterogeneity from alternate designs, such as studies with multiple interventions, are lacking.

Consideration of heterogeneity of treatment response is an especially salient issue for weight loss trials conducted in older adults. While advanced age and excess adiposity are well-recognized risk factors for chronic disease and disability [15], variability surrounding the risk-benefit of intentional weight loss in this population—particularly as related to disability risk—has stalled widespread clinical recommendation [16, 17]. Encouragingly, evidence from the majority of lifestyle-based randomized controlled trials (RCTs) of caloric restriction and exercise in older adults demonstrate *mean* improvement in physical function following clinically meaningful (5–10%) weight loss [15]; however, substantial variation in the magnitude of change exists, with a subset of participants inevitably experiencing a null or negative effect [18]. Better understanding of the extent and predictors of inter-individual variability in physical function treatment response in this population has the potential to optimize geriatric obesity treatment strategies, while also providing a clinically relevant platform upon which to refine modeling approaches designed to manage heterogeneity.

Several similarly designed RCTs testing the effects of caloric restriction and exercise on physical function among older adults with obesity were conducted over the past two decades at Wake Forest University and Wake Forest School of Medicine, providing an unique infrastructure to pool data to assess subgroup effects and account for inter-individual variability in treatment response [19–26]. Herein, we focus on change in gait speed, as it is arguably the most clinically relevant measure of physical function [27], and utilize a statistical modeling approach from a Bayesian framework to: 1) determine if there are inter-individual differences in gait speed as a result of randomization to caloric restriction, and 2) understand predictors of inter-individual variability in gait speed response, including demographic characteristics, health status, and exercise. We primarily hypothesize there will be an overall mean increase in gait speed because of caloric restriction, but that variability in gait speed change will also exist. We secondarily hypothesize that demographic characteristics, health status, and exercise assignment will interact with caloric restriction to influence gait speed mean and variance heterogeneity. Finally, as exploratory analyses we include continuous covariate data when available rather than dichotomized variables to determine if the subgroup differences persist using a linear model for the means and variances.

## Materials and methods

### Studies and participant descriptions

Relevant design characteristics of the included trials are summarized in Table 1. Briefly, individual participant data from eight RCTs conducted at Wake Forest University or Wake Forest School of Medicine and housed within the Wake Forest Older Americans Independence Center data repository were eligible for inclusion in the pooled analysis. Study-specific inclusion and exclusion criteria are summarized in S1 Table. Individual study lengths varied, but all studies assessed fast-paced gait speed before and five/six months after assignment to either a caloric restriction intervention (CR) with or without exercise or to a non-caloric restriction control condition (NoCR) with or without exercise. The Wake Forest Health Sciences Institutional Review Board approved secondary analyses pertaining to the pooled project (IRB#54086). As all data/samples were fully anonymized, the requirement for informed consent was waived under Exemption Category 4. The final sample (n = 1343) includes all randomized participants with baseline and follow-up gait speed data.

**Table 1:** Descriptive summary of randomized controlled trials included in the pooled analysis.

Study Acronym and NCT#	N (%♂; %Black)	Mean Age (years)	Health Status	Intervention (n)	Complete Gait Speed (n)
**ADAPT**	231	68	Overweight/Obese	CR (n = 65)	218
				AE (n = 57)	
**NCT00979043**	(29%; 21%)		OA	CR+AE (n = 54)	
				Control (n = 55)	
**APPLE**	33	70	Obese OA	CR (n = 15)	33
**NCT02239939**	(24%; 18%)			CR+VEST (n = 18)	
**CLIP**	267	67	Overweight/Obese	CR+AE (n = 95)	261
				AE (n = 86)	
**NCT00119795**	(34%; 17%)		CVD/METS	Control (n = 86)	
**I’M FIT**	109	70	Overweight/Obese	CR+RE (n = 55)	109
			At-risk for disability		
**NCT01049698**	(45%; 12%)			RE (n = 54)	
**IDEA**	376	66	Overweight/Obese	CR (n = 120)	351
**NCT00381290**	(30%; 17%)		OA	AE (n = 124)	
				CR+AE (n = 132)	
**INFINITE**	146	69	Obese	Low CR+AE (n = 52)	142
**NCT01048736**	(25%; 24%)			High CR+AE (n = 51)	
				AE (n = 43)	
**MEDIFAST**	82	70	Obese/At-risk for disability	CR (n = 43)	80
**NCT02730988**	(27%; 25%)			Control (n = 39)	
**SECRET**	99	67	Overweight/Obese	CR (n = 24)	89
				AE (n = 26)	
**NCT00959660**	(19%; 45%)		HFPEF	CR+AE (n = 25)	
				Control (n = 24)	

NCT# = clinicaltrials.gov identifier; n = sample size; %♂ = percent male; m/s = meters per second; OA = osteoarthritis; CVD/METS = cardiovascular disease or metabolic syndrome; HFPEF = heart failure with preserved ejection fraction; CR = caloric restriction; AE = aerobic exercise; RE = resistance exercise; VEST = weighted vest use during activities of daily living; SPPB: Short physical performance battery.

### Primary exposure measure: Caloric restriction

Arms within each study were collapsed into CR (n = 749) and NoCR (n = 594) categories based on whether CR to induce weight loss was specified in the original study protocol. As shown in Table 1, six studies randomized a subset of participants to traditional aerobic or resistance exercise (n = 854), with over half (n = 464) also receiving CR. Specifically, among thirteen study-specific interventions collapsed into the CR arm, five included participants randomized to CR only (n = 285), and six included participants randomized to CR combined with exercise (n = 464). Among ten study-specific arms collapsed into the NoCR arm, four included participants randomized to attention control (n = 204), and six included participants randomized to exercise only (n = 390).

### Primary outcome measure: Change in objectively measured fast-paced gait speed

Time recorded from the six-minute walk test (53% of the study sample) or fast-paced 400-meter walk test (47% of the study sample) was used to derive fast-paced gait speed at baseline and five/six month follow up. Gait speed, in general, is associated with survival among older adults [28], with long distance walk performance highly predictive of subsequent disability and death [29]. During the six-minute walk test [30], participants were asked to walk as far as they could around a circular track in six minutes. During the 400-meter walk test [31], participants were asked to briskly walk 10 laps of a 40-meter course and were given a maximum of 15 minutes to complete the test.

### Covariate measures

All studies captured self-reported demographic characteristics (age, sex, and race) and presence of select comorbidities [cardiovascular disease (CVD) and diabetes] via questionnaire at baseline. Standing height was measured using a clinical stadiometer and body mass was measured at baseline and five/six months follow up with a standard scale (with shoes and outer garments removed). Body mass index (BMI) was calculated as weight in kilograms divided by height in meters squared (kg/m^2^). Whole body fat mass was also measured in four studies (n = 958) using dual-energy x-ray absorptiometry (DXA) on the same machine [Hologic Discovery (Bedford, MA)] and following a standardized protocol [19, 22–24]. Lastly, high-sensitivity C-reactive protein (CRP; n = 1293) and interleukin-6 (IL-6; n = 1288) were measured on all available blood samples using standard methodology [32].

### Statistical analyses

Baseline data were analyzed using descriptive statistics, with means and standard deviations computed for continuous variables and counts and proportions for discrete variables, overall and by CR assignment. Crude unadjusted comparisons of CR assignment on changes in weight and gait speed were compared using independent t-tests. For our primary analysis, we modeled the impact of CR assignment (*x*_*cr*_ = 0 for control and 1 for CR) on both the five/six-month gait speed change mean response (*μ*_*i*_) and residual variability ($\sigma_{i}^{2}$) for the *i*th individual by fitting a linear model assuming changes in gait speed are normally distributed, $N(\mu_{i},\sigma_{i}^{2})$, where the mean:

$$\mu_{i}=\beta_{0,k}+\beta_{1}x_{cr}+\beta_{bl}w_{bl}$$ (1M)

in which *β*_0,*k*_ is a study-specific random intercept distributed $N(\beta_{0},\sigma_{s}^{2})$ where *β*_0_ is the control group mean and $\sigma_{s}^{2}$ is the study random effect, *β*_1_ is the mean CR treatment effect, and *β*_*bl*_ is the coefficient for baseline gait speed. Furthermore, we model the residual response variability $\sigma_{i}^{2}$ using:

$$\log\left(\sigma_{i}^{2}\right)=\phi_{0}+\phi_{1}x_{cr}$$ (1V)

such that differences in group response variability can be tested by comparing the parameter *ϕ*_1_ to 0, i.e., identifying whether the 95% posterior credible interval contains 0, and the CR groups’ variances are $\sigma_{cr=0}^{2}=\exp\left(\phi_{0}\right)$ and $\sigma_{cr=1}^{2}=\exp\left(\phi_{0}+\phi_{1}\right)$. Using the log-scale for modeling variance terms adds convenience because to the support for log(*σ*^2^) spans the full real line; therefore, all model parameters (***β***, ***ϕ***) can be fit using diffuse normal prior distributions (*N*(0,10)), and $\sigma_{s}^{2}$ with inverse-gamma(0.001,0.001), to minimize prior assumptions and to allow the data likelihood to dominate the posterior estimates.

Our secondary objective is to estimate and compare the impact of CR assignment and covariates, whether a continuous covariate *x*_*s*_ or discrete subgroup membership (*x*_*s*_ = 0 vs. 1), on changes in gait speed. We added terms for covariates and the treatment-covariate interaction such that:

$$\mu_{i}=\beta_{0,k}+\beta_{1}x_{cr}+\beta_{2}x_{s}+\beta_{3}x_{cr}x_{s}+\beta_{bl}w_{bl}$$ (2M)

where *β*_2_ is the coefficient for key covariates/subgroup membership, *β*_3_ quantifies the interaction, and all remaining model parameters remain the same as (1M). Furthermore, for the response variance we modified (1V) to include subgroup/covariate term *ϕ*_2_ and interaction term *ϕ*_3_ as follows:

$$\log\left(\sigma_{i}^{2}\right)=\phi_{0}+\phi_{1}x_{cr}+\phi_{2}x_{s}+\phi_{3}x_{cr}x_{s}.$$ (2V)

The combinations of Model (2M) and (2V) permitted separate estimates for treatment means and variances across continuous or discrete subgroups and tests for differences in treatment response variability across covariate levels by comparing *ϕ*_3_ to 0. Again, we fit noninformative diffuse prior distributions as described above using OpenBUGS.

Subgroups were defined by dichotomizing continuous baseline predictors: age ≥65 years, median BMI >33.3 kg/m^2^, sex-specific median percent body fat (male median: 32.1%, female median: 44.5%), IL-6 >2.5 pg/dL [33], CRP >3.0 mg/L [34] and gait speed <1.0 m/s [35]. Dichotomous baseline variables [sex (male/female), race (black/white), comorbidity status (CVD and diabetes: yes/no), and randomized assignment to exercise (yes/no)] were also analyzed as subgroups. Continuous covariates (age, BMI, percent body fat, log IL-6, log CRP, and baseline gait speed) were included in separate exploratory models as linear predictors for means and variances, with log adjustment for right skewed biomarkers. Mean models of gait speed response adjusted for study and baseline gait speed, except models in which baseline gait speed and gait speed subgroup were predictors, means were adjusted only for study. All associations and comparisons were determined based on whether the 95% Bayesian Credible Interval (BCI; analogous to a frequentist 95% Confidence Interval), overlaps the null value of 0, and all variability estimates are presented as standard deviations for ease of interpretation. Results focus on the effect of CR and whether CR interacts with subgroups or covariates. Summary data and frequentist comparisons were performed using SAS software, version 9.4 (SAS Institute, Cary, NC) using 2-sided hypothesis tests and assuming a Type 1 error rate of 0.05, while Bayesian models were created using OpenBUGS and executed in R using the package R2OpenBUGS.

## Results and discussion

### Baseline characteristics of study sample

Analyses were performed on a sample of 1343 participants, with baseline demographic and health characteristics presented in Table 2. Participants were older [mean (SD), 67.7 (5.4) years], predominantly female (69.8%), white (79.2%), and living with obesity [BMI: 33.9 (4.4) kg/m^2^]. Overall prevalence of self-reported CVD (4.9%) and diabetes (14.4%) was low and did not differ by CR assignment; however, most participants had higher levels of inflammation defined as IL-6 ≥2.5 pg/mL (55.0%) and CRP ≥3.0 mg/L (60.1%). Fast-paced gait speed was 1.2 (0.2) m/s, indicative of a well-functioning cohort at baseline [36], with only 174 (13.0%) presenting with gait speed below 1.0 m/s. Participant characteristics were mostly similar across treatment groups with respect to all baseline characteristics, except for a small difference in baseline body fat percentage [CR: 41.1 (7.0) % vs. NoCR: 40.2 (7.2) %, *p* = 0.04] among the subset of participants with baseline DXA (n = 958). Finally, most (63.6%) participants were assigned to some form of exercise, although proportions were comparable across groups (CR: 61.9% vs. NoCR: 65.7%, *p* = 0.16).

**Table 2:** Baseline characteristics of study sample, overall and by treatment group.

Variable	N^a^	Overall (N = 1343)	N^a^	CR (N = 749)	N^a^	NoCR (N = 594)
Age in years		67.7 (5.4)		67.6 (5.4)		67.7 (5.3)
≥65 years		932 (69.4)		527 (70.4)		405 (68.2)
Female Sex, n (%)		938 (69.8)		533 (71.2)		405 (68.2)
Black Race, n (%)	1328	276 (20.8)	742	161 (21.7)		115 (19.6)
Presence of Comorbidities, n (%)						
Cardiovascular Disease	1213	60 (4.9)	662	33 (5.0)	551	27 (4.9)
Diabetes	1329	191 (14.4)	739	100 (13.5)	590	91 (15.4)
Body Weight/Composition						
Weight in kg		93.7 (15.3)		93.8 (15.2)		93.7 (15.6)
BMI in kg/m^2^		33.9 (4.4)		34.0 (4.3)		33.7 (4.6)
≥ median BMI^c^		670 (49.9)		383 (51.1)		287 (48.3)
Percent body fat	958	40.7 (7.1)	537	41.1 (7.0)	421	40.2 (7.2)
≥sex-specific median^d^	958	480 (50.1)	537	281 (52.3)	421	199 (47.3)
Inflammatory Burden						
IL-6 in pg/dL	1288	3.7 (7.2)	720	4.0 (9.3)	568	3.4 (2.8)
≥2.5 pg/dL	1288	708 (55.0)	720	410 (56.9)	568	298 (52.5)
CRP in mg/L	1293	7.1 (9.3)	726	7.4 (9.3)	567	6.7 (9.2)
≥3.0 mg/L	1293	777 (60.1)	726	438 (60.3)	567	339 (59.8)
Fast Paced Gait Speed (m/s)		1.2 (0.2)		1.2 (0.2)		1.2 (0.2)
<1.0 m/s		174 (13.0)		91 (12.1)		83 (14.0)

Abbreviations: BMI: Body Mass Index; IL-6: Interleukine-6; CRP: C-reactive Protein

^**a**^Sample sizes provided only when they differ from column headers.

^b^Data are presented as mean (SD) or n (%).

^c^Median BMI was 33.3kg/m^2^.

^d^Median DXA-acquired percent fat mass was 32.1% for males and 44.5% for females.

### Overall effects on of CR assignment on weight loss and gait speed response

**Fig 1:**
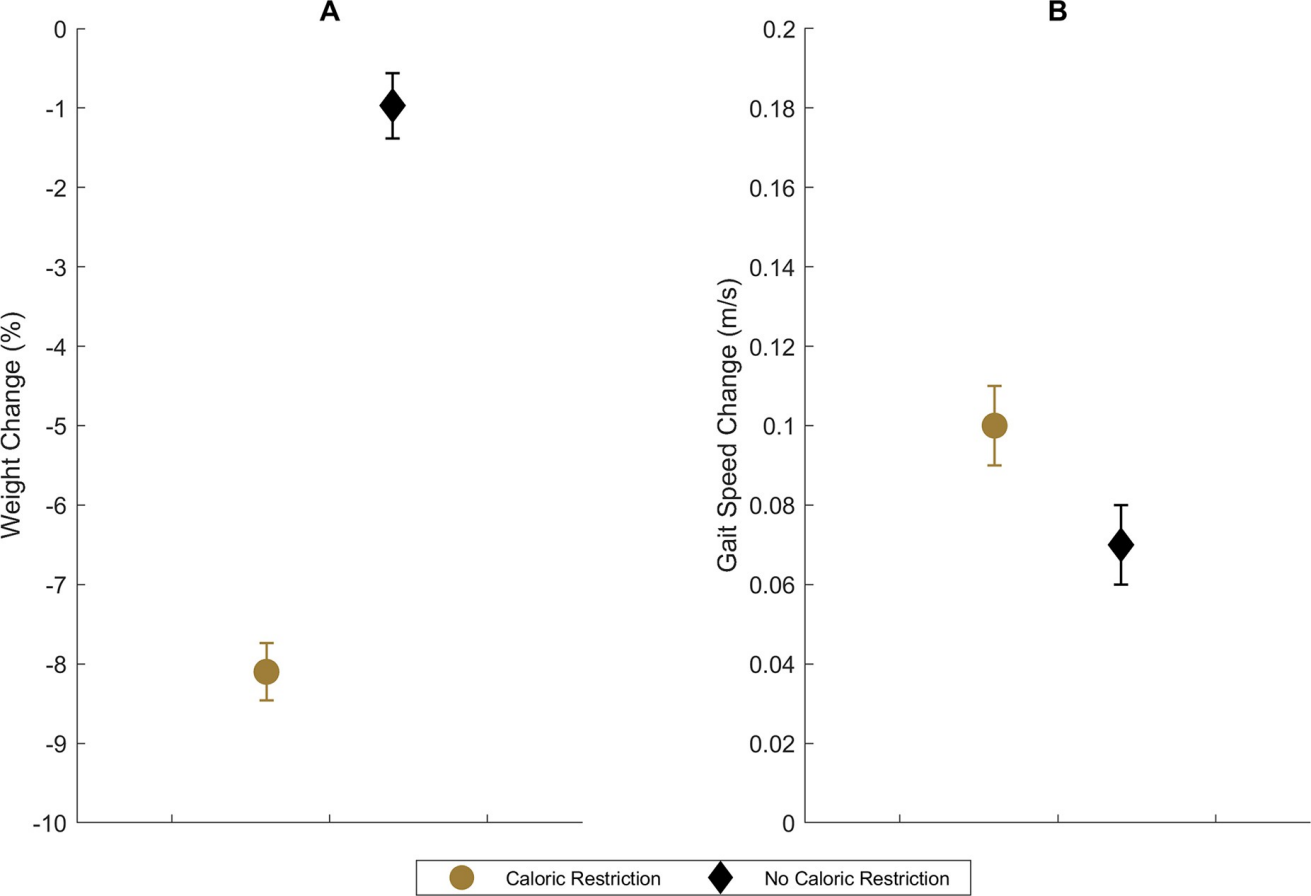
Crude six-month mean (95% Bayesian Credible Interval) change in percent weight (A) and fast-paced gait speed (B) by caloric restriction assignment group.

On average, participants assigned to CR significantly reduced their body mass more than those assigned to NoCR [absolute changes CR: -7.7 (5.8) kg vs. NoCR: -0.9 (3.5) kg, *p*<0.01; relative changes: CR: -8.1 (5.9) % vs. NoCR: -1.0 (3.7) %; *p*<0.01], with significant differences BMI change as well [CR: -2.8 (2.1) kg/m^2^ vs. NoCR -0.4 (1.3) kg/m^2^; *p*<0.01]. In unadjusted analyses, CR participants showed a significant increase in gait speed compared to NoCR [+0.10 (0.16) m/s vs. +0.07 (0.15) m/s, respectively, *p*<0.01] (see Fig 1). After adjustment for baseline gait speed and study, CR continued to yield a mean increase in gait speed [+0.021 m/s (95% Bayesian Credible Interval, BCI: 0.005, 0.037)], which was slightly attenuated compared to the unadjusted comparison. However, as illustrated in Fig 1, there were no differences in standard deviations between groups [CR: +0.143 m/s (95% BCI: 0.136, 0.151) vs. NoCR: +0.141 m/s (95% BCI: 0.133, 0.150)], indicating that although means differed by CR treatment, there was no observable heterogeneity of response variances due to overall CR assignment.

### Heterogeneity of gait speed change means and standard deviations across subgroups

Model adjusted outcomes that estimate treatment and subgroup heterogeneity across baseline demographic and health characteristic subgroups are presented in Table 3. Means are estimated from Model (2M) and standard deviations are estimated from Model (2V). Significant mean interactions between CR assignment and subgroup membership were observed for BMI (≥33.3 kg/m^2^) and IL-6 (≥2.5 mg/dL), while significant variance interactions were observed for age (≥65 years) and low gait speed (<1.0 m/s). Subgroups based on baseline BMI produced enhanced gait speed effects for CR among the higher BMI subgroup compared to the lower subgroup [CR benefit for BMI ≥ median: +0.039 m/s (95% BCI: 0.016, 0.061) vs. CR benefit for BMI < median: +0.004 m/s (95% BCI: -0.018, 0.027)], and participants with higher baseline IL-6 similarly experienced enhanced gait speed benefit from CR compared to the lower IL-6 subgroup [CR benefit for IL-6 ≥2.5 pg/mL: +0.041 m/s (95% BCI: 0.019, 0.62) vs. CR benefit for IL-6 <2.5 pg/mL: -0.001 m/s (95% BCI: -0.025, 0.024)]. Similarly, the subgroups of age and gait speed are associated with differences in standard deviations, with CR inducing increased variability among younger (<65 years) participants [CR vs. NoCR: +0.029 m/s (95% BCI: 0.009, 0.050)] vs. older (≥65 years) participants [CR vs. NoCR: -0.010 m/s (95% BCI: -0.024, 0.003)]; and reduced variability among lower baseline gait speed (<1.0 m/s) participants [CR vs. NoCR: -0.034 m/s (95% BCI: -0.075, 0.004)] compared to higher gait speed (≥1.0 m/s) participants [CR vs. NoCR: 0.010 m/s (95% BCI: -0.002, 0.022)]. Similar effects for the means were observed when modeling BMI and log IL-6 as continuous variables, where the added benefit of CR persisted with higher BMI and log IL-6 values; furthermore, the baseline gait speed interaction with CR persisted for the standard deviations using continuous baseline gait speed but not for continuous age (S2 Table).

**Table 3:** Subgroup mean and standard deviation estimates in gait speed change, presented by treatment group and after fitting an interaction term.

	Δ Gait Speed Mean Estimates (95% Bayesian Credible Interval)^c^				Δ Gait Speed Standard Deviation Estimates (95% Bayesian Credible Interval)^d^			
	NoCR	CR	CR–NoCR	Interaction	NoCR	CR	CR–NoCR	Interaction^b^
Age								
<65 years	0.067 (-0.002, 0.136)	0.081 (0.010, 0.149)*	0.014 (-0.016, 0.043)	0.011 (-0.024, 0.046)	0.128 (0.115, 0.143)*	0.158 (0.143, 0.174)*	0.029 (0.009, 0.050)*	-0.557 (-0.900, -0.221)*
≥65 years	0.041 (-0.027, 0.107)	0.065 (-0.002, 0.131)	0.024 (0.005, 0.044)*		0.147 (0.137, 0.159)*	0.137 (0.129, 0.146)*	-0.010 (-0.024, 0.003)	
Sex								
Male	0.086 (0.014, 0.155)*	0.090 (0.020, 0.159)*	0.004 (-0.028, 0.037)	0.026 (-0.011, 0.063)	0.156 (0.141, 0.173)*	0.159 (0.145, 0.175)*	0.003 (-0.018, 0.025)	0.021 (-0.314, 0.352)
Female	0.031 (-0.038, 0.097)	0.061 (-0.008, 0.127)	0.030 (0.012, 0.048)*		0.131 (0.123, 0.141)*	0.136 (0.128, 0.144)*	0.004 (-0.008, 0.017)	
Race								
White	0.056 (-0.014, 0.124)	0.071 (0.001, 0.138)*	0.015 (-0.004, 0.033)	0.032 (-0.005, 0.069)	0.143 (0.134, 0.153)*	0.147 (0.138, 0.156)*	0.004 (-0.010, 0.016)	-0.025 (-0.422, 0.364)
Black	0.011 (-0.062, 0.081)	0.058 (-0.013, 0.128)	0.047 (0.015, 0.079)*		0.129 (0.113, 0.148)*	0.130 (0.116, 0.146)*	0.002 (-0.022, 0.024)	
Cardiovascular Disease								
No	0.049 (-0.021, 0.118)	0.069 (-0.000, 0.138)	0.020 (0.004, 0.037)*	0.018 (-0.054, 0.090)	0.142 (0.134, 0.151)*	0.144 (0.136, 0.152)*	0.002 (-0.009, 0.014)	-0.424 (-1.258, 0.356)
Yes	0.016 (-0.074, 0.104)	0.055 (-0.026, 0.135)	0.039 (-0.032, 0.109)		0.143 (0.109, 0.194)*	0.117 (0.091, 0.151)*	-0.026 (-0.084, 0.024)	
Diabetes								
No	0.052 (-0.017, 0.120)	0.068 (-0.001, 0.134)	0.016 (-0.002, 0.033)	0.036 (-0.014, 0.084)	0.140 (0.131, 0.149)*	0.140 (0.132, 0.148)*	0.000 (-0.012, 0.012)	0.190 (-0.259, 0.655)
Yes	0.021 (-0.054, 0.093)	0.072 (-0.003, 0.146)	0.051 (0.005, 0.097)*		0.148 (0.128, 0.173)*	0.164 (0.142, 0.190)*	0.015 (-0.017, 0.049)	
Body Mass Index (using median split: 33.3 kg/^2^)								
<median BMI	0.067 (-0.002, 0.135)	0.071 (0.002, 0.138)*	0.004 (-0.018, 0.027)	0.034 (0.003, 0.066)*	0.144 (0.133, 0.156)*	0.146 (0.135, 0.157)*	0.002 (-0.014, 0.018)	0.037 (-0.271, 0.343)
≥median BMI	0.028 (-0.040, 0.096)	0.067 (-0.000, 0.134)	0.039 (0.016, 0.061)*		0.137 (0.126, 0.149)*	0.141 (0.131, 0.152)*	0.004 (-0.011, 0.020)	
Percent Fat Mass (using sex-specific median split: male = 32.1%, female = 44.5%								
<Sex-specific median	0.073 (-0.020, 0.165)	0.097 (0.004, 0.187)*	0.024 (-0.003, 0.051)	-0.001 (-0.039, 0.036)	0.143 (0.130, 0.159)*	0.142 (0.130, 0.155)*	-0.002 (-0.021, 0.017)	-0.120 (-0.493, 0.263)
≥Sex-specific median	0.054 (-0.039, 0.145)	0.077 (-0.016, 0.167)	0.023 (-0.003, 0.050)		0.143 (0.130, 0.159)*	0.133 (0.123, 0.146)*	-0.010 (-0.028, 0.009)	
Interleukin-6								
<2.5 pg/mL	0.068 (-0.005, 0.140)	0.068 (-0.006, 0.139)	-0.001 (-0.025, 0.024)	0.041 (0.009, 0.073)*	0.142 (0.130, 0.154)*	0.153 (0.142, 0.166)*	0.012 (-0.005, 0.029)	-0.212 (-0.527, 0.109)
≥2.5 pg/mL	0.029 (-0.043, 0.100)	0.069 (-0.003, 0.140)	0.041 (0.019, 0.062)*		0.137 (0.127, 0.149)*	0.134 (0.125, 0.144)*	-0.004 (-0.019, 0.011)	
C-Reactive Protein								
<3.0 mg/L	0.035 (-0.036, 0.105)	0.064 (-0.006, 0.134)	0.029 (0.009, 0.049)*	-0.018 (-0.052, 0.015)	0.137 (0.127, 0.148)*	0.132 (0.124, 0.142)*	-0.005 (-0.019, 0.009)	0.246 (-0.084, 0.572)
≥3.0 mg/L	0.065 (-0.007, 0.135)	0.076 (0.005, 0.147)*	0.011 (-0.016, 0.038)		0.144 (0.131, 0.159)*	0.157 (0.145, 0.172)*	0.013 (-0.006, 0.033)	
Baseline Fast Paced Gait Speed^a^								
≥1.0 m/s	0.041 (-0.023, 0.104)	0.062 (-0.002, 0.124)	0.021 (0.004, 0.038)*	0.014 (-0.044, 0.071)	0.137 (0.128, 0.146)*	0.146 (0.139, 0.155)*	0.010 (-0.002, 0.022)	-0.530 (-1.018, -0.062)*
<1.0 m/s	0.101 (0.025, 0.175)*	0.136 (0.064, 0.205)*	0.035 (-0.019, 0.090)		0.189 (0.161, 0.223)*	0.155 (0.134, 0.181)*	-0.034 (-0.075, 0.004)	

Abbreviations: NoCR: Non-caloric restriction arms; CR: Caloric restriction arms; kg: kilogram; m: meter; pg: picogram; mL: milliliter; mg: milligram: L: liter; s: second. *Denotes statistically significant (*p*<0.05).

^a^Baseline gait speed subgroup model was not additionally adjusted for baseline gait speed.

^b^Interaction term presented in log-adjusted scale.

^c^Means (95% BCI) estimated from Model (2M) of statistical analysis section. NoCR estimates correspond to *β*_0_ (row 1) and *β*_0_+*β*_2_ (row 2), CR estimates correspond to *β*_0_+*β*_1_ (row 1) and *β*_0_+*β*_1_+*β*_3_ (row 2), CR—NoCR correspond to *β*_1_ (row 1) and *β*_1_+*β*_3_+*β*_2_ (row 2). Interaction estimates correspond to *β*_3_.

^d^Standard deviations (95% BCI) estimated from Model (2V) of statistical analysis section. NoCR estimates correspond to exp(*ϕ*_0_) (row 1) and exp(*ϕ*_0_+*ϕ*_2_) (row 2), CR estimates correspond to exp(*ϕ*_0_+*ϕ*_1_) (row 1) and exp(*ϕ*_0_+*ϕ*_1_+*ϕ*_3_) (row 2), CR—NoCR correspond to exp(*ϕ*_1_) (row 1) and exp(*ϕ*_1_+*ϕ*_3_−*ϕ*_2_) (row 2). Interaction estimates correspond to *ϕ*_3_ (log-adjusted scale).

**Fig 2:**
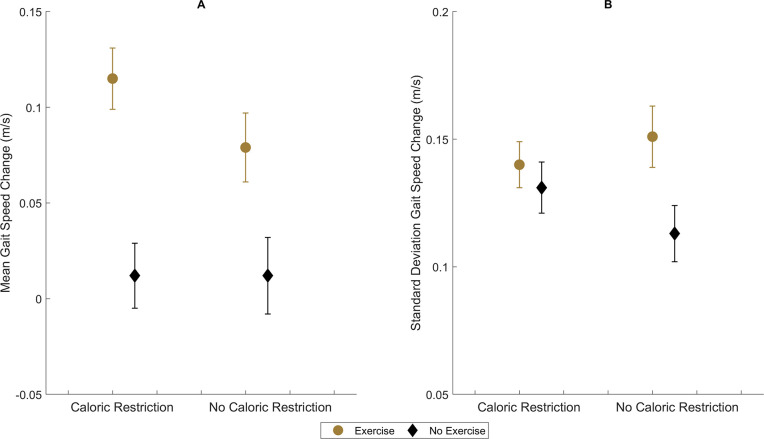
Effect of exercise subgroup membership on change in fast-paced gait speed mean (A) and variance (B) heterogeneity in response to caloric restriction. Presented as model-adjusted parameter means and 95% Bayesian Credible Intervals.

A significant interaction for mean gait speed change was observed between random assignment to exercise and CR [+0.037 m/s (95% BCI: 0.004, 0.070)]. As illustrated in Fig 2, participants who were not assigned to exercise had no difference in gait speed change according to CR assignment [-0.000 m/s (95% BCI: -0.026, 0.026)], but participants assigned to exercise plus CR had an added gait speed benefit compared to exercise plus NoCR [+0.037 m/s (95% BCI: 0.016, 0.057)]. Furthermore, exercise and CR significantly interact with regard to the standard deviations [-0.458 m/s (95% BCI: -0.783, -0.138)], leading to an increased SD attributable to CR among participants not assigned to exercise [+0.018 m/s (95% BCI: 0.002, 0.034)] but a non-significant reduction in the standard deviation among CR participants assigned to exercise [-0.012 m/s (95% BCI: -0.026, 0.003)].

## Discussion

The purpose of this study was to develop an approach to quantify the magnitude and predictors of variability in treatment response, specifically focusing on the clinical conundrum of physical function response to CR among older adults with obesity. Overall, we found that mean gait speed modestly and uniformly increased with CR (+0.02 m/s, compared to NoCR), which was largely driven by the CR-exercise interaction. The combination of CR and exercise had a significantly stronger and more consistent effect on mean gait speed change (+0.04 m/s) than exercise alone, and exercise had an interaction effect on CR treatment response variability. Additionally, those with a high BMI and elevated IL-6 experienced enhanced gait speed improvement in response to CR, while CR-subgroup interactions in gait speed variance were observed in age and low gait speed subgroups. Results of this study provide a statistical framework for examining inter-individual variability in treatment response and highlight a situation where identification of specific phenotypic characteristics (i.e., within this specific scientific domain: high BMI, and high IL-6) may help guide clinical decision making.

Consideration of inter-individual variability in treatment response, while often ignored in clinical trials, can offer insight into maximizing treatment efficacy. Indeed, previous clinical studies examining response variability have reported considerable treatment effect heterogeneity across various fields, such as exercise [6, 7] pharmaceuticals [4, 5] and dietary supplements [8, 9]. Within the field of geriatric weight management, our results suggest that older adults presenting with elevated BMI or inflammatory burden are more likely see CR-associated improvement in physical function in comparison to lower BMI/inflammatory burden counterparts. This finding is likely explained by the inflammatory nature of adipose tissue [37] and its association with impaired muscle fiber contractility [38]. Indeed, mobility impairment in older adults is associated with high BMI [39] and/or IL-6 [33] both of which can be reduced with CR [40]. Our observation that exercise paired with CR results in greater improvement in gait speed response as compared to CR alone, while notable, is not necessarily surprising. As structured exercise yields well-recognized improvement in muscle coordination and strength—even among older, sedentary adults [41, 42]—the combination of CR with exercise would be expected to further improve gait speed. What is surprising, however, is that CR plus exercise yields markedly and uniformly greater improvement in gait speed change (i.e. +0.04 m/s) versus *exercise alone*, and that CR alone had the same effect as the control condition (NoCR, no exercise) on gait speed change. These findings emphasize the need to combine therapies in order to maximize functional benefit and also temper the concern that CR alone (and presumably associated muscle loss [43]) exacerbates functional decline in older adults.

Findings surrounding variance interactions for exercise, age, and baseline gait speed indicate that CR can have significantly different effects on gait speed variability, which in the case of age and baseline speed may occur in the absence of a significant subgroup by treatment mean interaction. This observation is important to note, as exploration of subgroup effects for heterogeneous response in the absence of differential treatment effects on standard deviations has been criticized [14]; yet, our findings suggest there may be meaningful knowledge to be gained. For example, a situation could exist where differential achievement of clinically meaningful gait speed change (i.e. ≥0.05 m/s [44]) could occur within subgroups in the absence of a significant treatment difference in variances if the intervention induces different variability within subgroups. Furthermore, failure to quantify inter-individual variability in clinical trials may result in subgroups that experience a beneficial or negative treatment effect that differs from the overall study effect.

Future trials that use observed variability estimates without taking subgroups into account can yield biased and inaccurate power estimates. For an example from these analyses, a hypothetical trial of CR among women could use estimates from Table 3 to identify that the standard deviation of gait speed change among women is roughly comparable by randomization group (0.133 m/s), but this estimate is lower than the overall (men and women) standard deviation of change (0.142 m/s). A future trial using this information would require 110 observations per group rather than 128 per group for a 0.05 m/s difference using a two-sample t-test with 80% power, which could help improve study efficiency and decrease the budget. Collectively, these observations underscore the importance of testing for differences in variances (across treatment arms and within subgroups) when possible, to enhance the utility of clinical trial findings.

Strengths of this study include the uniquely large sample achieved by pooling individual level data from RCTs with similar major design elements and standardized protocols collecting gait speed data (including training/certification of functional assessors and use of standardized script language). In addition, heterogeneity among design aspects of the trials can be acknowledged as a limitation, particularly among differing CR targets and entry criteria, but it also broadens the generalizability of our findings and protects against over-interpretation of idiosyncratic results from any single study. Our subgroup analysis featured dichotomized and continuous predictors, with categorization based on medians to maximize power (BMI, body fat percentage) or empirical evidence (IL-6, CRP, gait speed). Although dichotomization of continuous variables is commonly criticized in the biostatistical literature [45], it is reassuring that we observed similar results using continuous linear predictors for both means and variances, with the sole exception of age. The use of studies performed at a single site could potentially limit the generalizability of the results due to circumstances and participants unique to the Winston-Salem area. Additionally, some individuals were participants in multiple trials; this could affect the assumption of independence between a small proportion of observations. Finally, while exercise assignment was shown to have heterogeneous effects with CR, unfortunately this analysis does not allow for consideration of exercise prescription characteristics, including frequency, intensity, type, and duration. We encourage future research efforts to confirm and extend this finding.

## Conclusions

In conclusion, this study examined gait speed response from CR and exercise trials among older adults and found uniformly increased gait speed with CR compared to NoCR, which was largely driven by exercise. Results also suggest that exercise combined with CR yields additional gait speed benefit for older adults compared with exercise alone, and that older adults with high baseline BMI and IL-6 are likely to experience enhanced gait speed change with CR. Furthermore, exercise treatment assignment as well as membership in age or baseline gait speed subgroups can yield differences in gait speed change variances. Our modeling approach provides a framework to detect novel sources of mean and variance response heterogeneity due to treatment interactions with covariates, while also creating an avenue for exploring heterogeneity in factorial design studies. We implore future clinical trials to consider mean and variance treatment response heterogeneity as a part of a pre-specified analytic strategy, when possible.

## Supporting information

S1 Table Inclusion and exclusion criteria for each randomized controlled trial included in the pooled analysis. (DOCX)

S2 Table Effects of WL and continuous characteristics on means and SDs. (DOCX)
