# Supplementary material for: Estimating heterogeneity of physical function treatment response to caloric restriction among older adults with obesity
Source: PLoS One. 2022 May 5;17(5):e0267779. doi: 10.1371/journal.pone.0267779 (PMC9070937; doi:10.1371/journal.pone.0267779)
Supplement: S1 Table — (DOCX) [file pone.0267779.s001.docx]

**S1 Table.** Inclusion and exclusion criteria for each randomized controlled trial included in the pooled analysis.

| **Study Acronym and clinicaltrials.gov identifier (NCT#)** | **Inclusion Criteria** | **Exclusion Criteria** |
| --- | --- | --- |
| **ADAPT**  **NCT00979043** | Age ≥60 years; BMI ≥28 kg/m^2^; knee pain on most days of the month; sedentary activity pattern with <20 minutes of formal exercise once weekly for the past 6 months; self-reported difficulty in at least one of the following activities ascribed to knee pain: walking one-quarter of a mile (3–4 city blocks), climbing stairs, bending, stooping, kneeling (e.g., to pick up clothes), shopping, house cleaning or other self-care activities, getting in and out of bed, standing up from a chair, lifting and carrying groceries, or getting in and out of the bathtub; radiographic evidence of grade I–III tibiofemoral or patellofemoral OA based on weight-bearing anteroposterior and sunrise view radiographs; willingness to undergo testing and intervention procedures. | Serious medical condition that prevented safe participation in an exercise program, including symptomatic heart or vascular disease (angina, peripheral vascular disease, congestive heart failure), severe hypertension, recent stroke, chronic obstructive pulmonary disease, severe insulin-dependent diabetes mellitus, psychiatric disease, renal disease, liver disease, active cancer other than skin cancer, and anemia; MMSE<24; inability to finish the 18-month study or unlikely to be compliant; inability to walk without a cane or other assistive device; participation in another research study; reported alcohol consumption of >14 drinks per week; ST segment depression of at least 2 mm at an exercise level of 4 METS or less, hypotension, or complex arrhythmias during a graded exercise test; inability to complete the protocol, in the opinion of the clinical staff, because of frailty, illness, or other reasons. |
| **APPLE**  **NCT02239939** | Age 65-79 years; BMI=30-40 kg/m2; weight<135 kg; self-reported physician diagnosed OA; no evidence of clinical depression or other contraindications for participation in voluntary weight loss; sedentary lifestyle (<30 minutes, 3 days/week of exercise); able to provide own transportation to study visits and intervention; not dependent on a cane or walker; willing and able to consume meal replacement products; not involved in another behavioral or interventional research study; approved for participation by Medical Director; willing to provide informed consent. | Weight loss or gain (±5%) in past 6 months; excessive alcohol use (> 14 drinks/week); smoker (>1 cigarette/day or 4/week within year); evidence of cognitive impairment (MoCA score <22) or difficulty with hearing or vision that would interfere with study participation; insulin-dependent or uncontrolled diabetes (FBG >125 mg/dl) or hypertriglyceridemia (TG>400 mg/dl); osteoporosis (T-score< -2.5 on hip or spine); hip fracture, joint replacement, or spinal surgery in past 6 months; hyperkyphosis or chronic severe back pain; uncontrolled hypertension (BP>160/90 mmHg) or abnormal kidney or liver tests per Medical Director discretion; self- reported hepatitis B or C; severe anemia (Hb<10 g/100 ml); uncontrolled endocrine/metabolic disease, neurological or hematological disease, fibromyalgia, chronic pulmonary disease, hyperparathyroidism, rheumatoid arthritis, unstable angina, MI, cardiac surgery within 3 months; cancer requiring treatment in past year, except skin cancers; regular use of medications that may influence body weight, hormones, weight loss medications, bone remodeling medication, prior use of medications that affect bone, anti-coagulants, or use of insulin; unable to tolerate vest run-in. |
| **CLIP**  **NCT00119795** | Community-dwelling men and women from the counties of interest; Age: 60-79 years; sedentary (fewer than 30 minutes of moderate physical activity each week that is structured and occurs in no less than 10 minute blocks); overweight or obese as defined by a BMI greater than 25 kg/m2; documented evidence of an MI, PCTA, chronic stable angina, or cardiovascular surgery (coronary artery or valvular heart disease) in the past 6-months or an ATP III diagnosis of the metabolic syndrome; disability defined as self-reported difficulty with walking ¼ mile, climbing stairs, lifting and carrying groceries, or performing other household chores such as cleaning and yard work; does not plan to move out of the county of residence for the duration of the study; willing and able to participate in all aspects of the trial; willing to give an informed consent and sign a Health Insurance Portability and Accountability Act of 1996 (HIPPA) authorization form. | Bipolar depression or schizophrenia (defined as self-reported treatment for these conditions), currently receiving lithium or neuroleptics; evidence of unstable angina, symptomatic congestive heart failure, or exercise induced complex ventricular arrhythmias; resting blood pressure >160/100 mmHg; fasting blood glucose > 140 mg/dL, diagnosis of type 1 diabetes, or diagnosis of type 2 diabetes and on insulin therapy; diagnosis of Parkinson’s disease, chronic liver disease (cirrhosis, chronic hepatitis, etc.), systemic rheumatic condition (rheumatoid arthritis, psoriatic arthritis, Reiter’s disease, systemic lupus erythmatosus), end stage renal disease or other systemic diseases or abnormal laboratory values which would preclude participants from safely participating in the protocol or impair their ability to complete the study; active treatment for cancer other than non-melanotic skin cancer; significant visual or hearing impairment that cannot be corrected and results in the inability to use the telephone or hear normal conversation; currently participating in or planning to participate in another medical intervention study; consuming more than 21 alcoholic drinks per week or alcoholism; unable to walk unassisted; unable to speak or read English; judged to be unsuitable for the trial for any reason by the clinic staff. |
| **I’M FIT**  **NCT01049698** | Age=65-79 years; BMI=27-34.9 kg/m^2^; no resistance training for past 6 months; normal cognitive function (MMSE >24); no contraindications for participation in weight loss or weight lifting exercise including severe arthritis or musculoskeletal disorders, knee or hip replacement or spinal surgery in past year; able to provide own transportation to study visits and intervention; not involved in any other research study or undergoing physical therapy; not dependent on a cane or walker; willing to provide informed consent; approved for participation by Principal Investigator and Medical Director. | Weight loss or gain (±5%) in past 6 months; body mass >136 kg (DXA limit); current smoker (no nicotine within past year) or evidence of alcohol or drug abuse; insulin dependent or uncontrolled diabetes (FBG >140 mg/dL); uncontrolled hypertension (BP>180/100 mmHg); abnormal kidney function or liver blood tests; serious conduction disorder (e.g., 3rd degree heart block), uncontrolled arrhythmia, or new Q waves or ST-segment depressions (>2 mm) on ECG; past or current cardiovascular disease, including uncontrolled angina or dysrhythmia, hypertrophic cardiomyopathy, congestive heart failure, PAD, stroke, history of myocardial infarction, use of defibrillator or major heart surgery, or deep vein thrombosis or pulmonary embolus; past or current respiratory disease (requiring steroid treatment or supplemental oxygen); past or current clinical diagnoses of neurological or hematological disease; use of any medications that could influence study variables (growth/steroid hormones, prescription anti-inflammatory medications, or beta blockers, Coumadin or any other blood thinner, including Plavix, Ticid, and Aggrenox); cancer requiring treatment in past 2 years, except non-melanoma skin cancers; clinically evident edema or anemia. |
| **IDEA**  **NCT00381290** | Ambulatory, community-dwelling persons; Age ≥ 55 years; grade II-III (mild to moderate) radiographic tibiofemoral OA or tibiofemoral plus patellofemoral OA of one or both knees; 27.0 ≤ BMI ≤ 40.5 kg/m2; a sedentary lifestyle, defined as not participating in a program that incorporates more than 30 minutes per week of formal exercise within the past 6 months. | Symptomatic or severe coronary artery disease; severe HTN; active cancer, other than skin cancer; anemia; dementia; liver disease; COPD; peripheral vascular disease; inability to walk without an assistive device; blindness; osteoporosis, ligament or cartilage damage from acute event; type 1 diabetes; type 2 diabetes on thiazolidinediones agents; patellofemoral OA without tibiofemoral OA; unwillingness or inability to change eating and physical activity habits due to environment; cannot speak and read; excess alcohol use ≥ 21 drinks per week; lives >50 miles from site or planning to leave area ≥ 3 months during the next 18 months; pacemaker, severe claustrophobia, defibrillator, implanted metal objects in leg, neurostimulator, magnetic aneurysm clip, any kind of metal implant or foreign metal objects in the body, such as bullets, shrapnel, metal slivers; significant cognitive impairment or depression diagnosis of dementia or a 3MSE score < 70, CES-D score > 17. |
| **INFINITE**  **NCT01048736** | Age ≥65 to <80 years; BMI=30-45 kg/m^2^; sedentary for past 6 months (<30 minutes, 3 days/week of exercise, including walking), not involved in any other research study or undergoing physical therapy; normal cognitive function (MMSE ≥ 24); no contraindications for participation in weight loss or exercise (e.g., severe arthritis or musculoskeletal disorders); able to provide own transportation to study visits and intervention; no drug abuse or excessive alcohol use (> 7 drinks/week); not dependent on a cane or walker; willing to provide informed consent; approved for participation by Medical Director. | Weight loss or gain (±5%) in past 6 months; body weight >136.4 kg (DXA limit is 300 lbs); smoker (no nicotine within past year); osteoporosis (T-score ≥ -2.5); abnormal kidney function tests; insulin-dependent or uncontrolled diabetes; uncontrolled hypertension (BP>200/110 mmHg); hypertriglyceridemia (TG>400 mg/dl); serious conduction disorder, uncontrolled arrhythmia, or new Q waves or ST-segment depressions (>2 mm) on ECG; past or current ischemic heart disease, angina, heart failure, peripheral artery disease, stroke, chronic respiratory disease, uncontrolled endocrine/metabolic disease, neurological or hematological disease, clinically evident edema; cancer requiring treatment in past 2 years, except non-melanoma skin cancers; clinical evidence of anemia; hip fracture, hip or knee replacement, or spinal surgery in past 6 months; regular use of medications that influence study variables (growth/steroid hormones, estrogen, anti-inflammatory, beta blockers, blood thinners). |
| **MEDIFAST**  **NCT02730988** | Age 65-79 years; BMI=30-42 kg/m2; confirmation of self-reported mobility disability, as assessed by phone screen/clinical staff; self-reported sedentary behavior; non-impaired cognitive function (MoCA>18); stability of residence for next 2 years; willing and able to follow dietary protocol; willing to provide informed consent; approved for participation by study physician; not involved in another behavioral or interventional research study; able to provide own transportation to study visits and intervention; not dependent on a cane or walker; no evidence of clinical depression, eating disorder, or other contraindications for participation in voluntary weight loss; English literacy. | Weight loss or gain (±5%) in past 6 months; prior bariatric surgery; multiple food allergies; difficulty with hearing/vision that interferes with study participation; excessive alcohol use (>14 drinks/week); smoker (>1 cigarette/day within year); Insulin-dependent or uncontrolled diabetes (FBG >140 mg/dl); uncontrolled hypertension (BP>160/100 mmHg); abnormal kidney tests (GFR<40, creatinine >2.0); regular use of medications that may influence body weight or composition; severe systemic disease (diagnosis of Parkinson’s disease, chronic liver disease, systemic rheumatic condition, gout, thyroid disease, end stage renal disease) or other systemic diseases/abnormal laboratory values which would preclude participants from safely participating in the protocol or impair their ability to complete the study; severe symptomatic heart disease or cardiovascular procedure within the past 3 months or history/current diagnosis/signs and symptoms of heart failure, with either reduced or preserved Left Ventricular ejection fraction; cancer requiring treatment in past year, except skin cancers; judged unsuitable for the trial for any reason by clinic staff. |
| **SECRET**  **NCT00959660** | Heart failure clinical score ≥3 or Rich Criteria (history of acute pulmonary edema, or occurrence of 2 or more of the following with subsequent improvement with diuretic therapy and with no other identifiable cause: dyspnea on exertion, paroxysmal nocturnal dyspnea, orthopnea, systemic edema, exertional fatigue); normal ejection fraction ≥50%; age >60 years; BMI >30 kg/m^2^. | Valvular heart disease as the primary etiology of CHF; significant change in cardiac medication <4 weeks; uncontrolled hypertension (controlled blood pressure is defined according to current JNC guidelines); uncontrolled diabetes; evidence of significant COPD (assessment may be made by PFT (pulmonary function test); recent or debilitating stroke; cancer or other non-cardiovascular conditions with life expectancy less than 2 years; anemia (<11 gms Hgb); significant renal insufficiency (creatinine >2.5mg/dl); uncontrolled major psychoses, depressions, dementia, or personality disorder; plans to leave area within 1 year; refuses informed consent; left ventricular ejection fraction < 50%; significant valvular heart disease; evidence of significant ischemia; 1 mm flat ST depression (confirm with echocardiogram wall motion); wall motion abnormality or decrease in global contractility; stopped exercising due to chest or leg pain or any reason other than exhaustion/fatigue/dyspnea; exercise SBP > 240 mmHg, DBP > 110 mmHg; unstable hemodynamics or rhythm; unwilling or unable to complete adequate exercise test; indwelling metal-containing prosthesis (orthopedic, valvular, other); pacemaker or defibrillator; history of welding occupation (ocular metal debris); uncontrollable claustrophobia; any other contra-indication to MRI; history of bleeding disorder; current anticoagulation; contraindication to stopping aspirin for 1 week; allergy to topical anesthetic. |
