# Supplementary material for: Estimating heterogeneity of physical function treatment response to caloric restriction among older adults with obesity
Source: PLoS One. 2022 May 5;17(5):e0267779. doi: 10.1371/journal.pone.0267779 (PMC9070937; doi:10.1371/journal.pone.0267779)
Supplement: S2 Table — (DOCX) [file pone.0267779.s002.docx]

**Table S2:** **Effects of WL and continuous characteristics on means and SDs**

|  | **Mean Model (2M) Parameter Estimates (95% Credible Interval)** | | | | **St Dev Model (2V) Parameter Estimates (95% Credible Interval)^b^** | | | |
| --- | --- | --- | --- | --- | --- | --- | --- | --- |
| **Baseline Covariate** | $\boldsymbol{\beta}_{\boldsymbol{0}}$**: Intercept** | $\boldsymbol{\beta}_{\boldsymbol{1}}$**: CR** | $\boldsymbol{\beta}_{\boldsymbol{2}}$**: Covariate** | $\boldsymbol{\beta}_{\boldsymbol{3}}$**: CR x Cov Intx** | $\boldsymbol{\phi}_{\boldsymbol{0}}$ **: Intercept** | $\boldsymbol{\phi}_{\boldsymbol{1}}$**: CR** | $\boldsymbol{\phi}_{\boldsymbol{2}}$**: Covariate** | $\boldsymbol{\phi}_{\boldsymbol{3}}$**: CR x Cov Intx** |
| Age in years | 0.050 (-0.018, 0.116) | 0.021 (0.005, 0.038)* | -0.004 (-0.006, -0.001)* | 0.000 (-0.003, 0.003) | -3.919 (-4.037, -3.801)* | 0.014 (-0.142, 0.172) | -0.008 (-0.033, 0.018) | -0.015 (-0.049, 0.015) |
| Body Mass Index (kg/m^2^) | 0.048 (-0.022, 0.118) | 0.021 (0.005, 0.037)* | -0.005 (-0.007, -0.002)* | 0.005 (0.001, 0.008)* | -3.938 (-4.053, -3.821)* | 0.053 (-0.103, 0.210) | -0.022 (-0.047, 0.004) | -0.002 (-0.039, 0.036) |
| Interleukin-6 (pg/mL) | 0.046 (-0.026, 0.114) | 0.023 (0.006, 0.039)* | -0.029 (-0.049, -0.010)* | 0.034 (0.010, 0.058)* | -3.939 (-4.061, -3.817)* | 0.034 (-0.129, 0.197) | 0.004 (-0.186, 0.194) | -0.234 (-0.483, 0.010) |
| C-Reactive Protein (mg/L) | 0.046 (-0.025, 0.115) | 0.023 (0.006, 0.039)* | -0.014 (-0.025, -0.003)* | 0.012 (-0.002, 0.027) | -3.953 (-4.071, -3.828)* | 0.039 (-0.127, 0.200) | -0.103 (-0.210, 0.007) | -0.091 (-0.229, 0.051) |
| Percent Fat Mass | 0.065 (-0.025, 0.155) | 0.023 (0.004, 0.042)* | -0.019 (-0.035, -0.004)* | 0.007 (-0.012, 0.025) | -3.901 (-4.043, -3.760)* | -0.077 (-0.265, 0.109) | -0.004 (-0.158, 0.142) | -0.140 (-0.344, 0.073) |
| Gait Speed (m/s)^a^ | 0.047 (-0.022, 0.116) | 0.022 (0.006, 0.038)* | -0.136 (-0.190, -0.082)* | -0.066 (-0.138, 0.005) | -3.930 (-4.047, -3.813)* | 0.037 (-0.120, 0.193) | -0.572 (-0.946, -0.210)* | 0.864 (0.319, 1.437)* |

Abbreviations: CR: Caloric restriction arms; kg: kilogram; m: meter; pg: picogram; mL: milliliter; mg: milligram: L: liter; s: second. ^*^Denotes statistically significant (*p*<0.05).

^a^Baseline gait speed subgroup model was not additionally adjusted for baseline gait speed.

^b^All standard deviation model parameter estimates presented in log-transformed scale.
